# Recent advances of long non-coding RNAs in control of hepatic gluconeogenesis

**DOI:** 10.3389/fendo.2023.1167592

**Published:** 2023-03-31

**Authors:** Zhe Wang, Jinyu Ma, Runze Wu, Yinghong Kong, Cheng Sun

**Affiliations:** ^1^ Key Laboratory of Neuroregeneration of Jiangsu and Ministry of Education, Co-innovation Center of Neurogeneration, NMPA Key Laboratory for Research and Evaluation of Tissue Engineering Technology Products, Nantong University, Nantong, China; ^2^ Department of Endocrinology, Changshu No.2 People’s Hospital, Changshu, Jiangsu, China

**Keywords:** long non-coding RNAs, hepatic gluconeogenesis, blood glucose, type 2 diabetes, PEPCK, G6Pase

## Abstract

Gluconeogenesis is the main process for endogenous glucose production during prolonged fasting, or certain pathological conditions, which occurs primarily in the liver. Hepatic gluconeogenesis is a biochemical process that is finely controlled by hormones such as insulin and glucagon, and it is of great importance for maintaining normal physiological blood glucose levels. Dysregulated gluconeogenesis induced by obesity is often associated with hyperglycemia, hyperinsulinemia, and type 2 diabetes (T2D). Long noncoding RNAs (lncRNAs) are involved in various cellular events, from gene transcription to protein translation, stability, and function. In recent years, a growing number of evidences has shown that lncRNAs play a key role in hepatic gluconeogenesis and thereby, affect the pathogenesis of T2D. Here we summarized the recent progress in lncRNAs and hepatic gluconeogenesis.

## 1 Introduction

Gluconeogenesis is an important biochemical process for maintaining glucose homeostasis in mammals, in which glucose is produced from non-carbohydrate substrates including lactate, pyruvate, propionate, glycerol, and amino acids. In general, gluconeogenesis will be activated when the blood glucose level is very low, often under fasting or starvation conditions. In the body of mammals, gluconeogenesis mainly occurs in the liver, though it may also take place in fewer amounts in the kidney and small intestine. In normal conditions, hepatic gluconeogenesis is finely tuned by hormones including insulin and glucagon and thus keeps blood glucose within physiological concentrations (1, 2). Insulin is a negative regulator of gluconeogenesis, while glucagon is a positive regulator (1, 2). Chronic ectopic increased gluconeogenesis is often associated with metabolic syndromes such as hyperglycemia, hyperinsulinemia, insulin resistance, and type 2 diabetes (T2D) (3). On the contrary, impaired gluconeogenesis may cause hypoglycemia and a shortage of energy supply, leading to dizziness, memory loss, or coma (4).

Human genomics research data revealed that 84% of the human genome could be transcribed, only 2% of which can encode proteins after transcription (5). Therefore, RNAs not only act as carriers of genetic information but also play a variety of regulatory functions. RNAs without protein coding capacity are called non-coding RNAs (ncRNAs), including microRNAs, long-chain noncoding RNAs (lncRNAs), small nucleolar RNAs (snoRNAs), and circular RNAs (circRNAs) (6, 7). Traditionally, noncoding RNA molecules with a length greater than 200 nt have been defined as lncRNAs (8). Accumulating evidence has shown that lncRNAs play a pivotal role in many cellular events, such as cell division, differentiation, migration, and apoptosis (9, 10). Each lncRNA has its own tissue-specific expression pattern, which defines its respective unique function (10, 11). For example, lncRNA-Bvht is a heart-associated lncRNA in mouse, which is enriched in embryonic stem cells (ESCs) and plays a key role in cardiomyocyte differentiation (12). Upon starvation or cold stimulation, some lncRNAs in adipose tissues are transcribed and participate in lipolysis and thermogenesis, which may hold therapeutic potential for treating metabolic diseases such as obesity and diabetes (13). Similarly, hepatocytes also have a unique lncRNA expression profile, which changes during liver development and regulates liver maturation (14). RNA-seq revealed 104 differentially expressed lncRNAs in the liver of T2D rats and bioinformatics analysis suggest these lncRNAs may correlate with the pathogenesis of T2D by affecting lipid metabolism, gluconeogenesis, inflammation, and/or endoplasmic reticulum stress (15). Moreover, metformin treatment induced a number of differentially expressed lncRNAs in the liver of mice, implying lncRNAs are involved in hepatic gluconeogenesis since the promising inhibitory effect of metformin on gluconeogenesis (16). LincIRS2 is an obesity-repressed lncRNA in the liver, its deficiency elevated blood glucose, promoted insulin resistance, and induced glucose output in mice (17). All these data strongly indicate the potential impacts of lncRNAs on hepatic gluconeogenesis. In this review, therefore, we summarized the recent progress regarding the roles of lncRNAs on hepatic gluconeogenesis. Meanwhile, we also discussed the therapeutic potentials of lncRNAs in ectopic gluconeogenesis-associated metabolic disorders such as insulin resistance and T2D.

## 2 Hepatic gluconeogenesis in health and diseases

As the main energy source of mammals, glucose is necessary for maintaining normal physiological functions for the central nervous system (CNS), retina, and red blood cells (18–20). Adults with normal body weight consume 160 g of glucose per day. Of them, the most glucose (∼120 g) was consumed by the brain (21). In addition to exogenous glucose obtained from food, most the endogenous glucose is stored as glycogen in various organs such as the liver and muscle. In humans, only approximately 15 g of glucose is available for consumption in extracellular fluid, termed blood glucose. The balance between endogenous glucose production and peripheral glucose uptake helps to maintain systemic glucose homeostasis (22). Under normal physiological conditions, the concentration of blood glucose is dynamically constant. Low blood glucose concentration causes fatigue, dizziness, and irreversible damage to the CNS (23, 24). Likewise, long-term high blood glucose is often associated with metabolic dysfunctions, including hyperglycemia, insulin resistance, and T2D. Therefore, blood glucose concentration is an essential indicator of glucose homeostasis, and the dynamic balance of exogenous glucose supply and endogenous glucose production is a key node for maintaining normal physiological blood glucose (25). The liver is an essential organ for keeping blood sugar balance. Hepatic glucose metabolism includes multiple pathways of decomposition and anabolism. Glycogen synthesis, glycogenolysis, gluconeogenesis, and glycolysis jointly determine the stability of blood glucose. Hepatic cells regulate the dynamic balance of glucose metabolism in response to environmental and nutritional changes in an autonomous or involuntary manner. Hepatic gluconeogenesis, as the main way of endogenous glucose production during starvation, plays an important role in maintaining blood glucose balance.

## 3 Overview of lncRNAs

### 3.1 Biogenesis and classification of lncRNAs

LncRNAs are mainly transcribed by RNA polymerase II and generally contain a 5’-m7G cap and a 3’-poly (A) tail (26, 27). lncRNAs were traditionally thought not to possess the ability to encode proteins; however, some lncRNAs, such as LINC00998, LINC00961, LINC00467, and LINC-PINT, have been found to encode specific small polypeptides (28–32). Human GENCODE database shows that more than 173,000 lncRNA transcripts were identified in the human genome (33). Of them, only a small part of lncRNAs has been functionally annotated, while a large number of lncRNAs remain to be determined (34, 35). Most lncRNAs are localized in the nucleus, while some lncRNAs are located in the cytoplasm or other sub-organelles, such as ribosomes and mitochondria (10). Compared with protein-coding genes, lncRNA genes are generally less conserved with lower expression profiles (36, 37). Despite sharing similar patterns of splicing, export, and quality control with mRNAs, most lncRNAs are trapped in the nucleus. In comparison to mRNAs, lncRNAs have fewer and longer exons, and for this reason, lncRNAs prefer the NXF1/NXT1 pathway for nuclear export (38). Furthermore, lncRNAs have lower splicing efficiency but higher splicing frequencies to increase their numbers (39).

Based on genomic location and functioning mechanism, lncRNAs are divided into five groups, intergenic lncRNAs (lincRNAs), intronic lncRNAs, sense lncRNAs, antisense lncRNAs, and bidirectional lncRNAs (40). A large number of non-coding regions are distributed among the coding regions of the human genome, accounting for 98-99%. The lncRNAs transcribed from these non-coding regions are called intergenic lncRNAs. LncRNAs transcribed from introns in the coding region are named intronic lncRNAs. Sense and antisense lncRNAs are transcribed from sense and antisense strands coding proteins, respectively. Currently, most studies are focused on lincRNAs and antisense lncRNAs. LincRNA shows functional importance due to its high active transcription, a certain degree of domain conservation, tissue-specific expression, and stability, whereas antisense lncRNA accounts for a large amount of human lncRNA (40).

### 3.2 Functioning mechanisms for lncRNAs

By various regulation models, lncRNAs can positively or negatively control coding gene expression, which could be occurred at different stages of eukaryotic gene expression (41). At the chromatin level, lncRNAs induce chromatin epigenetic modification to affect conformational structures of chromatin and thereby control gene expression. LncRNAs can regulate DNA methylation by recruiting DNMTs/TETs, sequestering DNMTs, or regulating the expression of DNMTs/TETs (42). Alternatively, lncRNAs act as decoys to sequester chromatin modifiers from specific genomic sites to induce chromatin remodeling (43). At the transcriptional level, lncRNAs can mediate gene silence or activation. For example, Airn, an antisense transcript of the Iinsulin-like growth factor 2 receptor (gf2r) gene, whose transcription causes Pol II to detach from the Igf2r promoter, resulting in transcriptional pause and gene silencing (44). At the post-transcriptional level, LncRNAs are involved in the post-transcriptional splicing of mRNAs. LncRNA Ctcflos mediates the selective splicing of PRDM16 to generate short isomers with a preference for thermogenesis, thereby promoting fat thermogenesis (45). Moreover, lncRNAs may also regulate gene expression by other means. For example, lncRNAs fold into higher-order structure to bind nucleoprotein and assemble ribonucleoprotein complex to participate in protein nuclear localization, or lncRNAs pair with other RNAs to recruit protein complexes or adsorb microRNAs to regulate gene silencing (46).

## 4 LncRNAs in hepatic gluconeogenesis

### 4.1 LncH19

H19 is the first lncRNA originally found in the liver extract, which is 2.3 kb in length and located on chromosome 11. After transcription, lncH19 is exported to the cytoplasm after a similar modification process as mRNAs, such as splicing, capping, and polyadenylation (47). It is enriched in embryonic stem cells and remains highly expressed in the adrenal gland, liver, and adipose tissue after birth (48). LncH19 loses the ability to translate into small peptides due to the special structure of the 5’-terminal (49). Therefore, lncH19 plays a role as an independent functional unit. A clinical study of obese women showed that human linH19 transcription levels were negatively correlated with body mass index (BMI) and homeostatic model assessment of insulin resistance (HOMA-IR) (50). Moreover, H19 has been shown to regulate glucose homeostasis and β cell function (51).

By RNA-seq, Goyal N et al. found that H19 was largely decreased in the liver of diabetic db/db mice, suggesting its potential role in glucose metabolism. In the following experiments, their functional studies showed that the knockdown of H19 stimulates gluconeogenic gene expression and hepatic glucose output in HepG2 cells and primary mouse hepatocytes (52). *In vivo* studies have shown that, in healthy mice, H19 absence results in dysregulated glucose metabolism including hyperglycemia, hyperinsulinemia, and intolerant insulin, glucose, and pyruvate tests (53). Mechanistically, H19 silencing increases the occupancy of P53 in the promoter of *Foxo1*, which promotes the transcription of *Foxo1*, a master regulator of gluconeogenic gene expression (52, 53). However, this view regarding the roles of H19 in gluconeogenesis has been challenged. Deng J et al. have shown that overexpression of H19 in a human liver cell line activates the gluconeogenic program, which is likely due to increased expression of HNF4α (54). Most recently, one report confirmed that overexpression of H19 in Hepa1-6 cells increases *Pck1* expression and gluconeogenesis by inducing the nuclear retention of FOXO1 (55). Also, in this study, H19 was identified as an imprinted gene for transducing hyperglycemia from paternal obesity to female offspring (55). Therefore, due to these inconsistent findings, the precise functions of H19 on gluconeogenesis are not clear. More studies are required to clarify this issue.

### 4.2 LncSHGL

Mouse lncSHGL is located on chromosome 17, and its homologous in humans is lncRNA B4GALT1-AS1. LncSHGL was low expressed in the liver of obese mice, and similarly, lncRNA B4GALT1-AS1 was significantly decreased in patients with nonalcoholic fatty liver disease (56). It has been shown that restoration of hepatic lncSHGL plays a beneficial role against hyperglycemia, insulin resistance, and hepatic steatosis in diabetic mice, while inhibition of lncSHGL worsens hyperglycemia and lipid deposition in livers (56). Mechanistic studies revealed that lncSHGL increases calmodulin (CaM) mRNA translation by recruiting heterogeneous nuclear ribonucleoprotein A1 (hnRNPA1). As a result, increased CaM suppresses gluconeogenic and lipogenic pathways in hepatocytes (56).

### 4.3 LncMEG3

Maternal expression gene 3 (*MEG3*) is an imprinted gene located on the human chromosome 14q32. It is the ortholog of the gene trap locus 2 (*Gtl2*) on mouse chromosome 12. LncMEG3 is generally considered to be a tumor suppressor, which is expressed in a variety of tissues and encodes lncRNAs associated with liver disease. Different from lncH19, the transcript of *MEG3* is positively correlated with obesity index and HOMA-IR in humans. In accordance, lncMEG3 is highly expressed in high-fat diet-induced obese mice and ob/ob mice (57). In primary hepatocytes, overexpression of lncMEG3 results in increased expression of *Foxo1*, *G6pc*, *Pck1*; meanwhile, insulin-stimulated glycogen synthesis was suppressed by lncMEG3 (57). These alterations could be reversed by lncMEG3 interference (57). In another study, lncMEG3 is found to be a glucagon-inducible lncRNA in mouse primary hepatocytes, where it interacts with miR-302a-3p as a competing endogenous RNA (ceRNA) (58). By this way, lncMEG3 increases CREB-regulated transcriptional coactivator 2 (CRTC2), which is a target of miR-302a-3p. Consequently, upregulated CRTC2 stimulates gluconeogenesis by activating the axis of PGC-1α/*Pck1*/*G6pc* in hepatocytes (58). Furthermore, as a ceRNA, miR-214 is another substrate of lncMEG3. In hepatocytes, lncMEG3 sequesters miR-214 to favor transcription factor 4 (ATF4) expression (59). ATF4 is capable of inducing the gluconeogenic program by affecting the transcriptional activity of FOXO1 (60). Therefore, lncMEG3 promotes gluconeogenesis in hepatocytes by targeting the axis of miR-302a-3p/CRTC2 or miR-214/ATF4.

### 4.4 LncBhmt-AS

Betaine homocysteine methyltransferase (BHMT) is an enzyme that catalyzes the synthesis of methionine from homocysteine and is associated with insulin resistance and diabetes (61, 62). *BHMT* is highly expressed in the liver of rodents, which may play a role in gluconeogenesis by interacting with L-serine dehydratase/L-threonine deaminase to affect the use of the amino acid for gluconeogenesis (63). Recently, a new lncRNA was discovered during fasting, which is an antisense RNA of *Bhmt*, therefore, named lncBhmt-AS (64). LncBhmt-AS is located on chromosome 13 in mice with 1464 bp in length. Deficiency of lncBhmt-AS restricts gluconeogenesis in primary hepatocytes and inhibits liver glucose production and gluconeogenic gene expression *in vivo* (64). In contrast, *Bhmt* overexpression restores gluconeogenesis induced by lncBhmt-AS knockdown (64). These evidences indicate that lncBhmt-AS plays an important role in regulating hepatic gluconeogenesis by targeting *Bhmt*.

### 4.5 LncGm10768

Gm10768 is a lncRNA specifically enriched in the liver. Cui et al. found an abnormal increase of lncGm10768 in mouse livers after fasting by RNA-seq (65). In addition, lncGm10768 is positively correlated with glucose production in mouse primary hepatocytes (65). Liver-specific knockout of lncGm10768 alleviates hyperglycemia and insulin resistance in db/db mice (65). LncGm10768 is localized in the nucleus and cytoplasm, therefore, lncGm10768 may regulate gene expression at both transcriptional and post-transcriptional levels. As endogenous competitive suppressors of microRNAs, LncRNAs can reverse gene silencing induced by microRNAs. miR-214 has a high affinity binding site with lncGm10768, and it decreases in response to lncGm10768 overexpression (65). As mentioned above, miR-214 can target and activate transcription factor 4 (ATF4) to inhibit the expression of *G6pc* and *Pck1* (59). Therefore, the positive impact of lncGm10768 on hepatic gluconeogenesis is likely due to the interaction with miR-214 (65). In this regard, lncGm10768 and lcnMEG3 play a similar role in hepatic gluconeogenesis by targeting miR-214, indicating different lncRNAs may have synergistic effects to regulate gluconeogenesis jointly.

### 4.6 LncGomafu

LncGomafu is a conserved lncRNA in mammalian species, which was localized in the nucleus in most cases. It has been well documented that lncGomafu plays a key role in neuronal development and involves in the pathogenesis of neuropsychiatric disorders (66, 67). Similar to lncMEG3, lncGomafu is highly expressed in the livers of ob/ob mice and mice on a high-fat diet (HFD) (68). Knockdown of lncGomafu in the liver inhibits hepatic glucose production and improves insulin sensitivity in obese mice. On the contrary, overexpression of lncGomafu increases blood glucose levels in lean mice. Mechanistically, lncGomafu competitively sponge miR-139 to increase *Foxo1* expression, increasing gluconeogenic gene expression and hepatic gluconeogenesis (68).

### 4.7 LncMALAT1

Metastasis associated lung adenocarcinoma transcript 1(MALAT1) is a conserved lncRNA located on human chromosome 11q13 with a length of 8.5 kb (26). LncMALAT1 is considered a biomarker for tumor diagnosis and has been proven to be involved in the regulation of several signaling pathways, including PI3K/AKT, NF-kB, MAPK/ERK (69). Knockdown of lncMALAT1 in HepG2 and FLC4 cells leads to increased glucose secretion and expression of gluconeogenic genes such as *G6pc* and *Pck1* (70). Meanwhile, this study revealed that the negative regulation of lncMALAT1 on gluconeogenesis is due to the upregulation of TCF7L2 (70). TCF7L2 has been shown to interact with the promoters of *G6pc* and *Pck1*, this interaction impedes the transcriptional activities of CREB/CRTC2 and FOXO1, thereby repressing gluconeogenic gene expression (71).

### 4.8 LncGm10804

LncGm10804 is highly enriched in high glucose-treated hepatocytes and livers of non-alcoholic fatty liver disease (NAFLD) model mice. Both *in vitro* and *in vivo* studies have shown that the knockdown of lncGm10804 reduces the expression of *Pck1* and *G6pc* in cultured hepatocytes and NAFLD mice. Meanwhile, lncGm10804 silencing alleviates hepatic steatosis and lipid accumulation by decreasing the expression of sterol regulatory element-binding protein-1c (SREBP-1c) and fatty acid synthase (FAS) in NAFLD mouse livers (72).

### 4.9 LincIRS2

LncRNA 4833411C07Rik was named LincIRS2 by Marta Pradas-Juni et al. for its location at 80 kb of 5’ *Irs2* (17). LincIRS2 is induced upon fasting or glucagon stimulation and responds to cAMP signaling (17), suggesting lincIRS2 might be involved in hepatic gluconeogenesis. Indeed, in lean mice, the knockdown of lincIRS2 in the liver induces enhanced blood glucose, insulin resistance and ectopic glucose output. Meanwhile, deficiency of *Mafg* in hepatocytes evokes a fasting-like gene expression profile as evidenced by elevated expression of *Fbp1*, *G6pc* and *Pck1* (17). Later, they found that MAFG controls the expression of lincIRS2 and thereby regulates glucose metabolism in the liver (17).

## 5 Conclusion and perspectives

**Figure 1:**
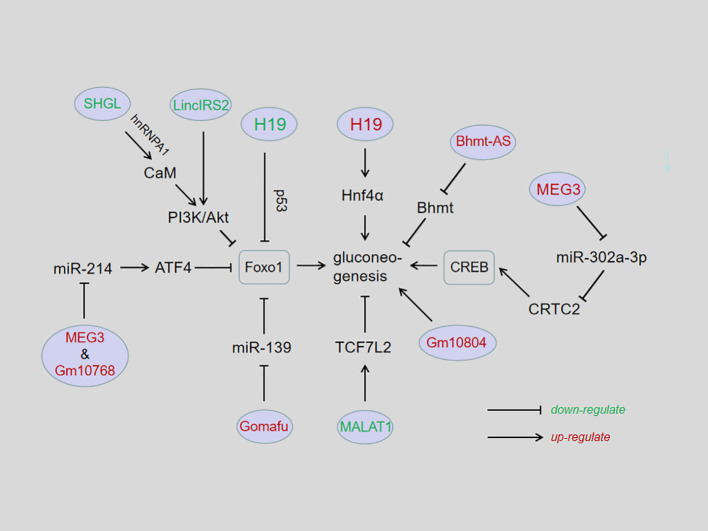
Influences of lncRNAs in hepatic gluconeogenesis. In general, lncRNAs affect hepatic gluconeogenesis in the following ways: lncRNAs directly regulate the expression of gluconeogenic genes, such as lncH19, lncSHGL, lncBhmt-AS, lcnMALAT1, lincIRS2, and lncGm10804; lncRNAs interact with miRNAs as competitive endogenous RNAs, such as lncMEG3/miR214; lncGm10768/miR214; lncRNAs act as microRNA molecular sponges to block subsequent pathways, such as lncH19/miRlet-7; lncMEG3/miR302-a-3p; lncGomafu/miR139; or lncRNAs bind to the promoter to affect gene transcription, such as lncH19/p53.

Hepatic gluconeogenesis is an essential bio-process to keep blood glucose in normal physiological scope. Dysregulated hepatic gluconeogenesis may course various metabolic disorders. For instance, ectopic upregulated gluconeogenesis is a causative factor for inducing hyperglycemia, hyperinsulinemia, insulin resistance, and T2D. In this regard, gluconeogenesis also is a target for developing anti-T2D drugs. Metformin is such an anti-T2D drug which has achieved great success in clinical. Therefore, in-depth studies on hepatic gluconeogenesis regulation and its molecular mechanisms are important for developing novel strategies for treating disorders induced by malfunctioned gluconeogenesis. The current evidences have shown that lncRNAs play a crucial role in hepatic gluconeogenesis, although just 9 lncRNAs have been examined in this field to date. Of note, these lncRNAs exhibited different functions on hepatic gluconeogenesis, lncH19, lncMEG3, lncGm10768, lncGomafu, and lncBhmt-AS function as positive regulators, whereas lncMALAT1 and lncSHGL act as negative regulators (**Table 1**). As for involved mechanisms, each lncRNA has its own working model (**Figure 1**).

**Table 1:** LncRNAs involved in control of hepatic gluconeogenesis.

LncRNAs	Location*	Expression	Function	Mechanisms	References
H19	The imprinted region of chromosome 11	Placenta, liver, adrenal gland	down-regulate	The interaction with p53 reduces the occupancy of p53 on *Foxo1* promoter and inhibits *Foxo1* transcription	(52, 53)
			up-regulate	It maintained *Hnf4α* hypomethylation level	(54)
			up-regulate	Induces *Foxo1* nuclear retention to increase gluconeogenic gene expression	(55)
B4GALT1-AS1(SHGL)	Chromosome 9 - NC 000009.12	Testicles, kidneys, liver	down-regulate	HnRNPA1 is recruited to increase calmodulin levels, activating Akt pathway and nuclear rejection of *Foxo1*	(56)
MEG3	Chromosome 14 -NC 000014.9	Placenta, adrenal gland, brain	up-regulate	By competitively binding to miRNA214 to reduce its repression on *Foxo1*	(59, 60)
			up-regulate	MEG3 adsorbs miRNA302A-3p to release locked CRTC2, resulting in synergistic effect with CREB	(58)
Bhmt-AS	Chromosome 5 - NC 000005.10	liver, kidney	up-regulate	BHMT-AS plays a key role in regulating hepatic gluconeogenesis by targeting *Bhmt*	(64)
Gm10768	Chromosome 19 -NC 000085.7 (Mouse origin)	liver	up-regulate	Competition with miRNA214	(65)
Gomafu	Chromosome 22 - NC 000022.11	Brain, adrenal glands, lymph nodes	up-regulate	Acting as a molecular sponge for miRNA139, the inhibition of *Foxo1* by miRNA139 was eliminated	(68)
MALAT1	Chromosome 11 - NC 000011.10	Bone marrow, thyroid and other 24 tissues	down-regulate	MALAT1 increases SRSF1 expression and activates the MTORC1-4EBP1 axis to regulate TCF7L2	(70, 71)
Gm10804	Chromosome 2-NC 000068.8(Mouse origin)	Kidney, placenta	up-regulate	Gm10804 overexpression is involved transcriptional activation of *Pck1* and *G6pc*	(72)
LincIRS2	Chromosome 8 - NC 000074.7 (Mouse origin)	Liver, stomach, mammary gland	down-regulate	LincIRS2 responds to cAMP signaling by inhibiting transcriptional activation of *G6pc*, *Pck1* and *Foxo1*	(17)

*All unlabeled loci are of human origin.

It should be mentioned that these gluconeogenesis-associated lncRNAs are mainly enriched in the liver. It is reasonable to predict that other tissues-derived lncRNAs may also be involved in hepatic gluconeogenesis, although no direct evidences support this prediction. It has been shown that lncRNAs are often carried by exosomes (73). Hence, lncRNAs derived from other tissues, such as muscle, pancreas, and fat, could be transferred into the liver *via* exosomes, where they might affect the gluconeogenic program. For this reason, exosomal lncRNA-mediated crosstalk between other tissues and hepatic gluconeogenesis could be investigated as a future direction. Therefore, we predicted that more lncRNAs potentially involved in the hepatic gluconeogenesis will be identified by RNA-seq technologies in non-liver tissues under stress, such as fasting or a high-fat diet. Engineered exosomes with specific lncRNAs with inhibitory effects on hepatic gluconeogenesis might hold great therapeutic potential for treating T2D. In addition, exosomal lncRNAs in blood might be diagnostic markers of dysregulated hepatic gluconeogenesis.

## Author contributions

ZW: Validation, writing-original draft. JM: Writing-original draft. RW: Writing-original draft. YK: Project administration, writing-review and editing. CS: Conceptualization, funding acquisition, writing-review and editing. All authors contributed to the article and approved the submitted version.
